# Protein Expression in the Nucleus Accumbens of Rats Exposed to Developmental Vitamin D Deficiency

**DOI:** 10.1371/journal.pone.0002383

**Published:** 2008-06-11

**Authors:** John McGrath, Takeshi Iwazaki, Darryl Eyles, Thomas Burne, Xiaoying Cui, Pauline Ko, Izuru Matsumoto

**Affiliations:** 1 Queensland Centre for Mental Health Research, The Park Centre for Mental Health, Richlands, Australia; 2 Department of Psychiatry, The University of Queensland, St Lucia, Queensland, Australia; 3 Queensland Brain Institute, The University of Queensland, St Lucia, Queensland, Australia; 4 Department of Pathology, The University of Sydney, Sydney, New South Wales, Australia; 5 Department of Psychiatry, Asahiyama Hospital, Futagoyama, Cyuo-Ku, Sapporo, Japan; Chiba University Center for Forensic Mental Health, Japan

## Abstract

**Introduction:**

Developmental vitamin D (DVD) deficiency is a candidate risk factor for schizophrenia. Animal models have confirmed that DVD deficiency is associated with a range of altered genomic, proteomic, structural and behavioural outcomes in the rat. Because the nucleus accumbens has been implicated in neuropsychiatric disorders, in the current study we examined protein expression in this region in adult rats exposed to DVD deficiency

**Methods:**

Female Sprague Dawley rats were maintained on a vitamin D deficient diet for 6 weeks, mated and allowed to give birth, after which a diet containing vitamin D was reintroduced. Male adult offspring (n = 8) were compared to control male (n = 8). 2-D gel electrophoresis-based proteomics and mass spectroscopy were used to investigate differential protein expression.

**Results:**

There were 35 spots, mapped to 33 unique proteins, which were significantly different between the two groups. Of these, 22 were down-regulated and 13 up-regulated. The fold changes were uniformly small, with the largest FC being −1.67. Within the significantly different spots, three calcium binding proteins (calbindin1, calbindin2 and hippocalcin) were altered. Other proteins associated with DVD deficiency related to mitochondrial function, and the dynamin-like proteins.

**Conclusions:**

Developmental vitamin D deficiency was associated with subtle changes in protein expression in the nucleus accumbens. Disruptions in pathways related to calcium-binding proteins and mitochondrial function may underlie some of the behavioural features associated with animal models of developmental vitamin D deficiency

## Introduction

Based on clues from epidemiology, we have proposed that low prenatal vitamin D may be a risk factor for later development of schizophrenia [1]. Many studies have shown that those born in winter and spring have a significantly increased risk of developing schizophrenia [2], and that those born at higher latitudes are also at increased risk of schizophrenia [3]. Given that vitamin D levels in the population fluctuate across the seasons and decrease across higher latitude [4], low prenatal vitamin D ‘fits’ these key environmental features and is therefore a plausible candidate risk factor for this disease. In order to explore the biological plausibility of this candidate, we have a rat model that we call the Developmental Vitamin D (DVD) model.

Rats exposed to low prenatal vitamin D have a broad range of neurobiological outcomes that are informative for schizophrenia research. Briefly, DVD-deficient neonates had larger lateral ventricles, increased cellular proliferation and reduced apoptosis, altered neurogenesis, reduced density of neurotrophin receptor (p75^NTR^), and reduced levels of nerve growth factor (NGF) and glial cell line-derived growth factor (GDNF) compared to controls [5]–[7]. As adults these animals had larger lateral ventricles and reduced NGF expression compared to controls [8]. Behaviourally, adult DVD-deficient rats were more active than controls (i.e. showing “hyperlocomotion”) [9], [10]. 5) DVD-deficient rats also have altered attentional processing indicated by impaired latent inhibition [11]. Some of the most robust and consistent findings in the DVD model have emerged in behavioural pharmacological studies. For example, we have shown that DVD-deficient rats have enhanced locomotion in response to the psychomimetic agents such as NMDA antagonist MK-801 [10], [12]. The DVD adult rat is also more sensitive to haloperidol, a dopamine (DA) D2 receptor antagonist [10], that is a widely used antipsychotic medication used to treat schizophrenia. Dysfunction of DA signalling has been strongly implicated in the pathogenesis of schizophrenia [13]. Dopamine projections involve a range of cortical and subcortical regions, however its role in the nucleus accumbens has been of particular interest with respect to neuropsychiatric disorders [14]–[16]. In the nucleus accumbens, dopamine influences the integration of inputs from the ventral hippocampus, the amygdala, and the prefrontal cortex. Grace and colleagues have suggested that dopamine may modulate a range of limbic and cortical functions relevant to the pathophysiology of schizophrenia via the nucleus accumbens [17].

Previously we explored the genomic and proteomic characteristics of frontal cortex and the hippocampus in the adult DVD rat [18], [19]. In particular, a proteomic study based on two cortical regions of DVD-deficient rats (frontal cortex and hippocampus), identified 36 dysregulated proteins. These proteins are associated with several biological pathways including oxidative phosphorylation, cytoskeleton maintenance, calcium homeostasis, chaperoning, synaptic plasticity and neurotransmission. A computational analysis of these data revealed that many of the proteins dysregulated in the DVD model have also been shown to be altered in schizophrenia post-mortem brain studies [18].

In order to further explore the impact of DVD on brain function, we undertook a proteomic study of the nucleus accumbens.

## Methods

To obtain vitamin D_3_ depletion, female Sprague-Dawley rats (Herston Animal Facility, Queensland, Australia) were kept on a vitamin D deficient diet (Dyets Inc., PA, USA). Animals were housed on a 12-h light/dark cycle (lights on at 06:00 h) using incandescent lighting, to avoid ultraviolet radiation within the vitamin D_3_ action spectrum. These conditions were maintained for six weeks prior to mating and throughout gestation. Control animals were kept under similar conditions except they received a vitamin D replete diet (Dyets, PA, USA and Specialty Foods, WA, Australia) and were housed under standard lighting conditions. Dams (and corresponding litters) were placed under control conditions for the remainder of the experiment. The male pups were weaned on postnatal day 21 and housed in groups of 3–6. Female rats were not used in these experiments, because the estrous cycle can introduce variability in protein expression in the region of interest [20], [21]. All procedures were performed with approval from the Queensland University Animal Ethics Committee, under the guidelines of the National Health and Medical Research Council of Australia.

### Sample Preparation

Tissue was sampled from 8 control and 8 DVD deficient male adult offspring. These animals were from four separate litters per experimental group. The nucleus accumbens (NAc) were sampled from all animals according to boundaries determined from a standard rat brain atlas [22]. Sample preparation and two dimensional gel electrophoresis (2DE) methodology was performed according to Alexander-Kaufman *et al* [23]. Briefly, 0.04 g–0.07 g of crude fresh frozen NAc tissue was placed in Buffer 1 (7M Urea, 2M Thiourea, 1% C7bZO, 40 mM Tris). Sample suspensions were sonicated 3×10 sec at 40% intensity and centrifuged at 14,000× *g* for 20 min at 15°C. The supernatant was reduced and alkylated in 5 mM tributylphosphine (TBP) and 1M acrylamide monomer at room temperature for 2 hr. The reaction was quenched by adding 10 mM DTT followed by 20 mg citric acid to adjust pH to approximately pH 6.0. Samples were precipitated using 5 volumes of room temperature acetone for 10 min and centrifuged at 3,500× *g* for 15 min at 15°C. The pellet was air dried for 5 min and resuspended in Buffer 2 (7M urea, 2M thiourea, 1% C7bZO).

### Two Dimensional Gel Electrophoresis

Protein concentration was determined by the Bradford method [24]. The 8 DVD deficient and 8 control sample tissues were used to perform duplicate 2DE analyses, providing a total of 16 gels for each group. Pre-cast immobilised pH gradient strips (IPG, 11 cm, pH 3–10, Proteome Systems, North Ryde, Australia) were passively rehydrated in 200 µg sample protein extract for 6 hr at room temperature. In the first dimension, rehydrated strips were focused using an *ElectrophoretIQ^3^* isoelectric focusing system (Proteome Systems) for a total of 120 kVh. IPG strips were reduced, alkylated and detergent exchanged using SDS equilibration buffer (Proteome Systems, 20 min) and loaded onto pre-cast SDS-PAGE gels (*GelChip™* 2D, 6–15%, 10×15 cm; Proteome Systems) for second dimension molecular mass separation using the *ElectrophoreticIQ^3^* system (50mA/gel, 15°C for 90 min).

### Image acquisition and analysis

Gels were fixed in solution containing 25% (v/v) methanol and 10% (v/v) acetic acid for 1 hr and stained using colloidal Coomassie Blue for spot visualization. Gels were scanned using a transmissive, flatbed scanner (UMAX) and analyzed using *Phoretix 2D Expression* software (Nonlinear Dynamics, Newcastle-upon-Tyne, UK). Following background subtraction and volume normalization of all gels, average gels were created for each group to assist comparison and reduce within group variations. Averaging parameters were set at 70%, therefore for a protein spot to appear in the averaged gel it must be present in 70% of all gels within a group. One-way Analysis of Variance (ANOVA) statistical tests were used to reveal statistically significant protein expression differences between the two groups (p<0.05). Protein spots that were significantly altered were excised for identification by mass spectrometry.

### Mass Spectrometry

Excised spots were washed in 50 mM ammonium bicarbonate/acetonitrile (60∶40 solution) for 1 hr at room temperature. Spots were dried in a Vacuum Concentrator (Eppendorf, Hamburg Germany) for 25 min and rehydrated at 4°C in tryptic digest solution (10 ng/µl porcine sequencing grade trypsin (Promega) in 50 mM NH_4_HCO_3_) for 1 h. Remaining tryptic digest solution was removed and gel pieces suspended overnight at 37°C in 50 mM NH_4_HCO_3_.

For protein identification, approximately 0.8 µl of the peptide mixture was spotted onto a target plate and covered with the same volume of matrix solution (α-cyano-4-hydroxy cinnamic acid (Sigma), 8 mg/ml in 70% (v/v) acetonitrile/1% (v/v) formic acid) and allowed to air dry. In several cases, peptides were concentrated and desalted using C_18_ Perfect Pure Tips (Eppendorf). Tips were activated with acetonitrile and washed with 5×10 µl of 1% (v/v) formic acid. The peptide mixture was then bound and aspirated 5 times through the column and bound peptides washed with 5×10 µl of 1% formic acid. Peptides were eluted in 0.8 µl of matrix solution directly onto a MALDI-TOF target plate. Peptide mass maps of tryptic peptides were generated by matrix assisted laser desorption/ionisation time-of-flight mass spectrometry (MALDI-TOF MS) using an Applied Biosystems Q-STAR Pulsar with MALDI source (APAF, University of Sydney). Mass calibration was performed using trypsin autolysis peaks, 2211.11 Da and 842.51 Da as internal standards.

### Database searching and secondary analyses

Data generated from peptide mass mapping (PMM) of each spot were used to perform searches of the SWISS-PROT, NCBI and TrEMBL databases using the programs Aldente (www.expasy.ch) and MASCOT (www.matrixscience.com). Identifications were based on the observed pI and M_r_ (kDa) of the matched protein, the number of matching peptide masses and the total percentage of the amino acid sequence that those peptides covered, in comparison to other database entries. Generally, a peptide match with at least 30% total sequence coverage was required for confidence in identification, but very low and high mass protein, and those resulting from protein fragmentation may not always meet the criterion. For searches performed using MASCOT, E-value and Score, as well as matching peptides and sequence coverage, were used to determine matches.

Although many proteins were found to be significantly altered in DVD-deficient adults, the fold-change level was insufficient for confirmation analysis by western blot. Bioinformatics was employed as secondary analyses. Depending of the size of the fold changes, we planned to use western blot and/or bioinformatics as secondary analyses. Significantly dysregulated proteins were examined in bioinformatic pathways analysis (Ingenuity Pathway Analysis [IPA]; Ingenuity Systems, Mountain View, CA). This manually-curated database builds hypothetical networks based on the candidate proteins and other potentially associated proteins in the database. For each pathway, scores are calculated as the negative base-10 logarithm of the *P* value, indicating the likelihood that the dysregulated proteins would be found in a given network by chance. Finally, proteomic analyses are prone to Type I errors. While we did not adjust the *p* value for the number of comparisons undertaken, we report the false discovery rate, which provides an estimate of the proportion of significantly different proteins (p<0.05) that may be truly null [25], [26].

## Results

**Figure 1:**
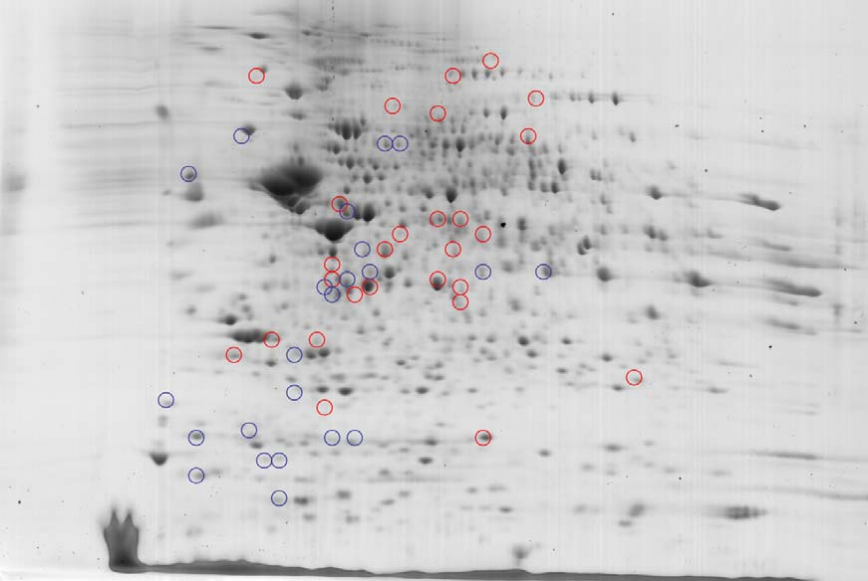
Altered proteins in the nucleus accumbens in adult DVD-deficient and control male rats. Red circles indicate increased spots, blue circles indicate spots reduced in the average gel in DVD animals.

We identified 637 spots in DVD deplete samples and 655 spots in Control samples (see Figure 1 for a representative gel). These spots were matched, normalized and quantified. For each animal, two gels were averaged. There were 35 spots that were significantly altered between the two groups, 22 were down*-*regulated and 13 up-regulated (Table 1). These spots were mapped to 33 unique proteins. The fold changes were uniformly small, with the largest FC being −1.67 (MEPD). Over half (27 of 49) of the significantly dysregulated spots that were identified as a known protein had a fold change of less than 1.3. Based on a False Discovery Rate of 28%, we predict that nine of these spots would be false positives.

**Table 1:** Summary of differentially expressed protein spots in nucleus accumbens tissue from adult DVD-deficient and control rats

Fold change	p value	Protein	Uniprot Accession Number	Mowse score	Molecular weight	pH
		**Calcium Binding Proteins**				
1.14	0.031	Calreticulin CALR	P18418	114	47966	4.33
-1.25	0.050	Calbindin (calretinin) CALB2	P47728	156	31384	4.94
-1.18	0.045	Calbindin CALB1	P07171	69	29975	4.71
1.17	0.032	Hippocalcin HPCA	P84076	79	22413	4.87
		**General cellular metabolism proteins**				
1.47	0.026	L-lactate dehydrogenase B chain LDHB	P42123	119	36589	5.70
1.23	0.004	Aldose reductase ALDR	P07943	154	35774	6.26
-1.11	0.014	crystallin, mu CRYM	Q9QYU4	96	33533	5.34
-1.09	0.030	Malate dehydrogenase, cytoplasmic MDHC	O88989	69	36460	6.16
-1.35	0.030	Glycerol-3-phosphate dehydrogenase [NAD+], cytoplasmic GPDA	O35077	141	37428	6.16
-1.17	0.043	Glutathione S-transferase P GSTP1	P04906	78	23424	6.89
		**Mitochondria Proteins**				
-1.54	0.041	Hexokinase 1 HXK1	P05708	76	102342	6.29
-1.35	0.048	Dynamin 1-like protein DNM1L	O35303	106	83856	6.64
-1.12	0.046	Ubiquinol-cytochrome-c reductase complex core protein 1, UQCR1	Q68FY0	145	52815	5.57
-1.14	0.033	Isocitrate dehydrogenase (NAD) subunit alpha, mitochondrial IDH3A	Q99NA5	113	39588	6.47
1.16	0.037	IDH3A	Q99NA5	117	.	.
-1.37	0.011	NADH dehydrogenase 1 alpha subcomplex subunit 10, NDUAA	Q561S0	219	40468	7.64
-1.10	0.039	Pyruvate dehydrogenase E1 component subunit beta, ODPB	P49432	95	38957	6.20
-1.18	0.033	Voltage-dependent anion-selective channel protein 2 VDAC2	P81155	59	31726	7.44
		**Signal transduction and MAP-related proteins**				
-1.28	0.007	Mitogen-activated protein kinase 1 (ERK-2; MAPK 2) MK01	P63086	57	41249	6.50
-1.24	0.009	Serine/threonine-protein phosphatase 2A catalytic subunit beta isoform PP2AB	P62716	107	35552	5.21
1.23	0.036	Guanine nucleotide-binding protein G(I)/G(S)/G(T) subunit beta 1 GBB1	P54311	64	37353	5.60
-1.15	0.031	Guanine nucleotide-binding protein G(I)/G(S)/G(T) subunit beta 2 GBB2	P54313	74	37307	5.60
-1.24	0.029	Dynamin 1 DYN1	P21575	152	95867	6.32
-1.67	0.047	Thimet oligopeptidase MEPD	P24155	112	78264	5.54
-1.22	0.006	Dihydropyrimidinase-related protein 2 (DRP-2; CRMP-2) DPYL2	P47942	101	62239	5.95
1.17	0.038	DPYL2	P47942	53	.	.
1.24	0.007	DPYL2	P47942	85	.	.
-1.21	0.024	Syntaxin-binding protein 1 STXB1	P61765	91	67526	6.49
-1.25	0.036	Adenosylhomocysteinase SAHH	P10760	93	47507	6.07
1.31	0.013	Ras-related protein Rab-3C RAB3C	P62824	64	25856	5.10
1.14	0.003	Beta-synuclein SYUB	Q63754	56	14495	4.48
1.17	0.013	Myosin light polypeptide 6 MYL6	Q64119	54	16964	4.46
		**Proteins not otherwise classified**				
1.48	0.011	Eukaryotic initiation factor 4A-II IF4A2	Q5RKI1	90	46373	5.33
1.19	0.001	Glyceraldehyde-3-phosphate dehydrogenase G3P	P04797	78	35805	8.14
-1.15	0.047	ADP-ribosylation factor 1 ARF1	P84079	128	20684	6.32

**Figure 2:**
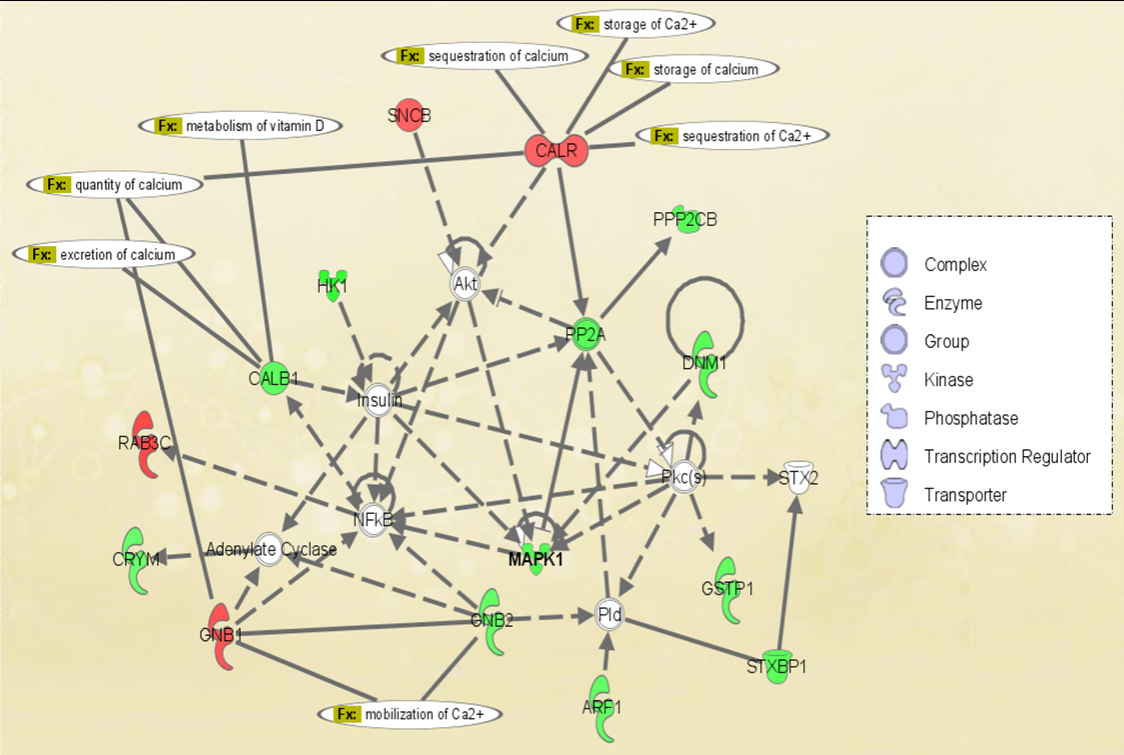
Network analysis of the proteins assembled by Ingenuity Pathway Analysis. Proteins symbols in red were up-regulated, while green were down-regulated. Hub proteins not significantly dysregulated in the study are shown as clear. The proteins with calcium-related functions are annotated (Fx).

Ingenuity Pathway Analysis identified two major networks; (a) Cellular Movement, cellular assembly and organization, cell signaling; and (b) Protein synthsis, RNA Post-transcriptional Modification, Cancer. These pathways had scores of 32 and 29 respectively (both highly significant). Figure 2 shows the first pathway, annotated for functions related to calcium buffering. Several of the components of the network converge on MAPK1, a MAP kinase that serves to integrate multiple biochemical signals (previously known as ERK2), that was found to be significantly down-regulated in the proteomics study.

## Discussion

Developmental vitamin D deficiency is associated with a subtle alteration in the expression of protein involved in functions related to calcium binding proteins, and mitochondrial functioning.

Calcium binding proteins have been of interest to schizophrenia research for some time [27], in particular with respect to the expression in cortical GABAergic interneurons [28]. This study found that four calcium binding proteins were significantly altered in the nucleus accumbens of the adult DVD-deficient rat (calbindin, calbindin2, hippocalcin and calreticulin). Calcium binding proteins are central to a wide range of cellular functions, of which calcium sequestration and buffering are particularly important for neurons. Amongst other functions, it is thought that this family of diffuse cytoplasmic proteins provides a ‘sink’ allowing cells to rapidly buffer intracellular calcium after actions potentials, thus allowing the cell to rapidly repolarise for further firing. Calbindin is strongly induced by vitamin D [29], and thus it is feasible that the reduction in this protein may be a consequence of the early life reduction in vitamin D. The potential links between vitamin D and neuronal calcium binding proteins has been noted in a recent review article [30]

Two members of the dynamin family (dynamin 1 and dynamin 1-like proteins) were also significantly down-regulated. These proteins are essential for clathrin-mediated endocytosis, a role of particular importance in neurons for neurotransmitter release [31]. Apart from this function, these proteins are essential for the insertion of dopamine receptor 2 (DRD2) into the nonsynaptic membrane of dopaminergic neurons [32]. Syntaxin-binding protein 1 was also significantly down-regulated in the DVD-deficient rats. These findings suggest that proteins involved in SNAP and SNARE mediated vesicle release may be disrupted in the DVD model. In addition, six mitochondrial proteins were down-regulated in DVD-deficient rats (NDUAA, UQCR1, ODPB, IDH3A, HKK1, VDAC2). These findings suggest that cellular energics may be altered in this brain region in the DVD-deficient rat. Curiously, it has recently been shown that dynamin 1-like protein is also important for mitochondrial fission and general morphology [33].

It is known that down-regulation of calcium binding proteins shift essential calcium buffering requirements to the mitochondrial compartment, which, in turn, leads to compromised mitochondrial energy production [34]. With respect to the nucleus accumbens, calcium-binding proteins such as calbindin are often used to demarcate discrete neuroanatomical boundaries, inferring that these proteins confer selective functional properties to these cells [35]. Compromised calcium buffering in the nucleus accumbens could disrupt adaptive and goal-directed behaviors. In the core region of the nucleus accumbens, neurons have dense spines, which may reflect the degree of synaptic plasticity required for the integrative function of cells in this region [36]. Many of the dopaminergic cells in this region also express neuropeptides such as bradykinan, neurotensin and substance P. The protein with the greatest fold change in this study was thimet oligopeptidase (down-regulated 1.67), which is involved in the degradation of these small proteins [37]. Neuropeptides such as neurotensin can indirectly influence dopaminergic transmission in the nucleus accumbens via glutamate and GABA-ergic mediated processes [38]. Thus, these neuropeptides are of interest as potential targets for novel antipsychotic agents [39], [40]. Curiously, it has been shown that calcium concentration is an important modulator of thimet oligopeptidase activity [41], thus the disruption of this protein may also be down-stream consequence of altered calcium buffering.

With respect to schizophrenia, several of proteins identified in this study have also been reported to be disrupted in post-mortem brain tissue from patients with schizophrenia. A range of studies (proteomics, genomics, gene association studies) have linked schizophrenia with alterations in malate dehydrogenase cytoplasmic (MDHC, now known as malic enzyme 2) [42]–[47]. Similarly, mitogen-activated protein kinase 1, significantly down-regulated in this study, has been found to be down-regulated at both the mRNA and protein levels in post-mortem schizophrenia brain tissue (thalamus) [48]

Like the previous proteomic study [18], we found no alterations in proteins directly associated with DA signaling. This suggests that baseline DA signaling may be normal in this model and abnormalities only become unmasked in the presence of drugs that alter DA/glutamate balance in the brain. It is conceivable that a slight reduction in calcium buffering proteins may affect the ability of neurons within the nucleus accumbens to repolarise in response to psychomimetic agents. Alternately, any reduction in cellular energetics within this region may delay the integration of cortical and/or sub-cortical inputs.

Disappointingly, we found no overlap with the proteins identified in the current study versus those in the previous publication [18]. However, the previous study was based on cortical and hippocampal tissue from adult female animals, whereas the current study was based on tissue from the nucleus accumbens in adult male animals. Interpretation of the current study is also limited because of the lack of immunoblot confirmation of the differentially expressed spots. The small fold changes found in the study, while statistically significant, were too low to be reliably confirmed via immunoblot [49]. Based on the behavioural findings in DVD-deficient rats, there is a case to explore proteomic dysregulation in rats after exposure to drugs known to disrupt dopaminergic and glutaminergic pathways. For example, we have shown that while habituated DVD rats have normal locomotion activity in the open field at baseline, they have pronounced hyperlocomotion after exposure to MK-801 [10], [12]. We plan to explore these issues in future experiments.

In conclusion, developmental vitamin D deficiency is associated with subtle changes in a range of proteins in the nucleus accumbens. These findings suggest that pathways involved in calcium binding and mitochondrial function may underpin the behavioural features associated with this particular animal model of schizophrenia. Combined with other experimental findings, the current study lends further credibility to the notion that developmental vitamin D deficiency impacts adversely on normal brain development [30].
